# Femtosecond laser-assisted keratopigmentation outcomes for pure cosmetic purposes

**DOI:** 10.1016/j.ajoc.2025.102297

**Published:** 2025-03-07

**Authors:** Jorge Alio, Azad Sanginabadi, Amir Theodore Hojabr, Behzad Jafari

**Affiliations:** a Division of Ophthalmology, Universidad Miguel Hernández, Alicante, Spain; b Vissum Alicante, Spain; c Department of Optometry, Iran University of Medical Sciences, Tehran, Iran; d Hs2AOphthalmogical Division, France; e Farabi Eye Hospital, Tehran University of Medical Sciences, Tehran, Iran

**Keywords:** Keratopigmentation, Corneal tattoo, Femtosecond laser–assisted corneal tattoo, Cosmetic cornea, Eye color, Corneal opacity

## Abstract

**Purpose:**

To assess the visual performance and complications of Femtosecond laser-assisted keratopigmentation for pure cosmetic purposes in a large consecutive series of cases.

**Observations:**

This prospective study included 166 eyes that underwent pure keratopigmentation between 2021 and 2023 by one expert surgeon. Demographic data, preoperative examination findings, postoperative outcomes, and complications were collected and analyzed. Data were collected on preoperative and postoperative visual acuity, refraction, intraocular pressure, light sensitivity, endothelial cell count, visual field, and aberrometry. Preoperative and postoperative values were compared to determine any changes. There was no statistically significant difference (p > 0.05) in visual acuity, Mean deviation(MD) and IOP at 12 months after surgery. The presence of horizontal and vertical coma and trefoil aberrations, as well as spherical and HOA (Higher Order Aberration) errors, does not have statistically significant meaning. The mean endothelial cell count decreased from 2393.29 ± 123.69 cells/mm^2 preoperatively to 2308.58 ± 126.64 cells/mm^2 postoperatively. The contrast sensitivity has decreased across all spatial frequencies for mesopic conditions, and the decreases at 1.5 Cycles Pre Degree (CPD) and 18 CPD for photopic conditions were statistically significant. However, it still remains within the normal range.

**Conclusions and importance:**

Pure cosmetic keratopigmentation seems to be a safe and effective surgical technique for improving the cosmetic appearance of the eyes. The technique showed no significant impact on visual acuity, intraocular pressure, visual field. However, slight decreases in contrast sensitivity and endothelial cell count were observed, which should be considered when discussing potential risks and benefits with patients.

## Teaser

Précis:Pure Keratopigmentation can be further investigated as a promising and safe way to change eye color in the future.

## 1 Introduction

Keratopigmentation is a novel technique that involves the injection of pigments into the cornea to improve visual acuity and cosmetic appearance in patients with traumatic eye and iris defects and, corneal opacities.^1^^,^^2^. While this technique has shown promising results in patients with ocular pathology, there has been relatively little research on the use of keratopigmentation as a purely cosmetic procedure in healthy eyes.^3^.

Pure cosmetic keratopigmentation involves the injection of pigments into the corneal stroma for the sole purpose of improving the color or appearance of the eye.^3^. This technique has garnered increasing attention in recent years, particularly among individuals seeking alternative to traditional surgical procedures such as Laser iris depigmentation or cosmetic iris implants. However, the safety and efficacy of pure cosmetic keratopigmentation have not yet been fully explored, and there is a need for further research to better understand the risks and benefits of this approach.^4^^,^^5^.

This study aims to evaluate the outcomes of intrastromal keratopigmentation performed for pure cosmetic purposes, specifically focusing on the effects on visual acuity, endothelial cell count, visual field aberrometry, and contrast sensitivity.

## 2 Patients and methods

**Study Design**: This study was a prospective, consecutive non-randomized, non-comparative clinical study designed to evaluate the safety, efficacy and complications of pure cosmetic keratopigmentation in healthy eyes. This work complies with the ethical standards of the relevant national and institutional committees on human experimentation and with the Helsinki Declaration of 1975, as revised in 2008." and "The authors assert that all procedures contributing to this work comply with the ethical standards of the relevant national and institutional guides on the care and use of laboratory animals. The authors affirm that all procedures conducted as part of this study align with the ethical standards outlined in the relevant national and institutional guidelines.

Prior to the surgery, all patients were adequately informed about the procedures and potential risks of the operation and subsequently provided their informed consent by signing a consent form. In all stages of project initiation, planning and surgeries were fully supervised by Professor Jorge Alio.

**Inclusion Criteria**: Participants were eligible for inclusion if they were healthy adults with normal ocular anatomy and without any ocular or systemic diseases that could affect corneal health or vision.

**Exclusion Criteria**: individuals with blind or poorly functioning eyes, as well as those with disfiguring conditions that affect the lens, pupil, or cornea. Additionally, patients with visual impairments related to ocular surface disorders, abnormal corneal topography and tomography, angle of kappa is greater than 4 mm, history of femto-LASIK or incision lenticule extraction(Smile), radial keratectomy(RK) thin corneas(500μm<), endothelial cell count less than 2000 cells/mm^2^, dry eyes, and dermatological disorders such as atopy, as well as those who reported sensitivities, were also excluded from the study.

### 2.1 Surgical procedure

All surgeries have been performed by an expert surgeon (A.T. H). Before the operation, the patient's eyelids were cleaned using Blephaclean wipes twice a day for three days leading up to the surgery, with one wipe used per eye each time. 4 minutes before the surgery, a drop of 0.5 % tetracaine was administered to both eyes. During the surgical procedure, the patient was positioned under the VisuMax® femtosecond laser and methylene blue was used to mark the center of the cornea. The ICR® program was then used to create a tunnel for the insertion of intracorneal rings, which are used in the treatment of keratoconus. The surgical parameters included an external diameter of 9.5mm, internal diameter of 5.0 mm–6.50 mm depth incision of 190μm, and one radial incision at 270° and to reduce the need for manual tunnel opening, we are employing a large docking lens and selecting a flat keratometry (38.00 D) to ensure that the tunnel is created close to the limbus. But for eyes with large white to white, we had to do some of it manually. The tunnel was dissected using the lamellar dissector (KTP corneal dissector; Epsilon, Irvine, CA), the peripheral dissected cornea was injected with the appropriate pigment Biochromaeyes (BIOTIC Phocea, France). using a 27-gauge cannula.

**Post-surgically**, the patients were treated by topical drop ciprofloxacin 0.3 % (Ciloxan, Alcon, Fort Worth, TX, USA)one drop every 4 hours for a week and betamethasone (Betnesol N, GSK pharmaceutical Ltd) one drop every 4 hours tapered over one month and preservative-free artificial tear drops (Artelac Advanced, Bausch & Lomb) 4 time in day for 3 months.

**Outcome Measures**: The primary outcome measures were changes in visual activity, visual field, endothelial cell loss, aberrometry, contrast sensitivity, light sensitivity, and slit lamp examination (corneal clarity, epithelial staining, inflammation, uniformity and design or pigment leakage) following the intervention. These measures were assessed at baseline and at,^3^^,^^6^, and 12 months' post-intervention. To measure patient satisfaction, we asked five general questions both over the phone and during the last visit. The questions included options for response ranging from "Very satisfied" to "Satisfied," "Neutral," "Dissatisfied," and "Very dissatisfied."

**Visual Acuity**: Uncorrected and Best Corrected Visual acuity (UCVA, BCVA) was measured using an Early Treatment of Diabetic Retinopathy Study (ETDRS) charts units for statistical analysis.

**Visual Field and Intraocular pressure:** Visual field testing was performed using automated perimetry (Humphrey Field Analyzer) with the 24-2 SITA Fast program. Intraocular pressure(IOP) was performed with the Topcon CT-1P (Topcon, Tokyo, Japan). Mean deviation Index (MDI) and Intraocular pressure(IOP) were calculated for analysis.

**Endothelial Cell Count**: Endothelial cell density was measured Specular microscopy was conducted by a skilled optometrist(A.S.), utilizing a semiautomatic non-contact Konan Noncon Robo Pachy SP-9000 specular microscope (manufactured by Konan Medical Inc., Hyogo, Japan). The procedure involved capturing three digital photographs of the central cornea, specifically within a 3 mm radius from the center. The analysis of the digital images was carried out only when the image resolution was clear, and the boundaries of the cells were distinguishable (area of the endothelium measured 400 × 220 mm). The preoperative and postoperative endothelial cell densities divided by preoperative density. We measured the changes in the Mean ± SD during the first, second and third trimesters, alongside the endothelial cell count (ECC), coefficient of variation (CV), and hexagonality (HEX).

**Aberrometry**: Wavefront aberrations were measured using the Pentacam HR (Oculus Optikegrate). The vertical and horizontal Coma and trefoil, Spherical aberration and high order aberration(HOA)were calculated for analysis.

**Contrast Sensitivity**: Contrast sensitivity was measured using the CVS1000 contrast sensitivity test (VectorVision, Greenville, SC) under photopic (85 cd/m2) and mesopic (3 cd/m2).

#### 2.1.1 Pigments

The pigments (BIOTIC Phocea, France) used in this study have medical device certification (EPT 0477.MDD.17/2601.4) are specifically designed for keratopigmentation in ocular pathologies, which are biocompatible and adapted to the pH of the cornea with high stability and low toxicity. These pigments, called Biochromaeyes and made in France, consist of various amounts of propanediol, and micronized mineral pigments (color index: 77,007, 77,491, 77,499, 77,492, 77,288, and 77,891). To ensure compliance with regulations, the pigments were marked with a CE certification (EPT 0477.MDD.17/2601.4). The pigments were tailored for existing eyes (color from human nature) according to a pre-determined list of colors. In most cases, computer simulations were performed using the patient's photo and the selected color to determine the most suitable option in consultation with the group.

## 3 Statistical analysis

To describe quantitative data, measures such as the mean, standard deviation, median, and range were used. Paired t-tests or their non-parametric equivalent, the Wilcoxon test, were used to examine and compare the means of variables in different groups, depending on the distribution of the variable. All analyses were performed using version 26 of the SPSS software. A p-value of less than 0.05 was considered statistically significant.

## 4 Results

In this study, 83 patients (166 eyes) consecutively underwent bilateral pure keratopigmentation, of whom 22 % were male and 78 % were female. The mean age of the evaluated patients was 33.05 ± 6.29 years, ranging from 24 to 50 years. After surgery, they underwent assessment at three intervals: 3, 6, and 12 months.

**Fig. 1:**
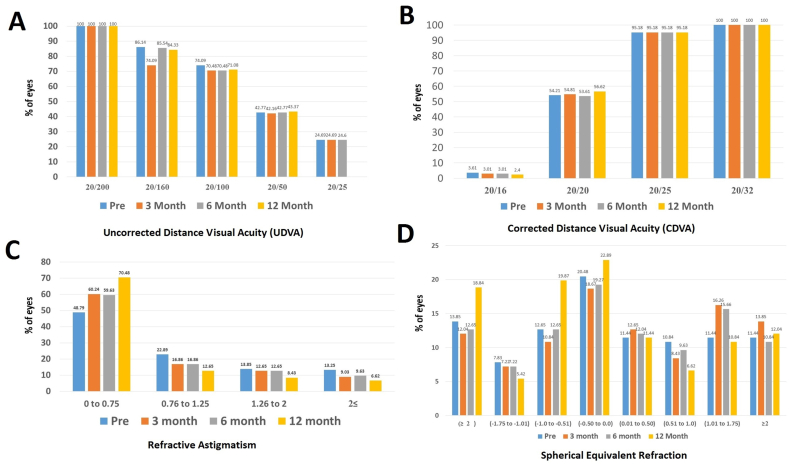
Visual and refractive outcomes after pure cosmetic keratopigmentation during follow up. (A) uncorrected distance visual acuity(CDVA). (B) corrected distance visual acuity(UDVA). (C) stability of spherical equivalent refraction. (D) stability of refractive cylinder value.

In Fig. 1(C and D), the examination of patients' refraction information and comparison in different follow-ups was addressed. Statistically, The average spherical equivalent in eyes that have experienced a slight increase in the negative range (P = 0.041). The mean of the cylinder also had a significant decrease both in month 12 compared to before surgery (P < 0.001). However, changes in the average of SE did not show a significant difference in follow-ups.

In Fig. 1(A and B), the UDVA and CDVA data were examined and compared in different follow-ups. Fig. 1(C and D): Refraction Data and Comparison in Various Follow-Ups.

No significant statistical difference was observed.

In Table 1, the information on the aberrometry of patients was presented and compared across different follow-ups. The average spherical aberration showed a significant difference compared to before surgery at months 6 (P = 0.042).

**Table 1:** Aberrometry data of patients and comparison in different follow-ups.

Parameters	Mean ± SD	Median (Range)	p-within
Vertical coma.Pre	0.42 ± 0.11	0.42 (0.1,0.65)	**0.012**
Vertical coma.3mo	0.42 ± 0.11	0.45(0.1,0.65)	
Vertical coma.6mo	0.42 ± 0.11	0.45 (0.1,0.65)	**0.063**
Vertical coma.12mo	0.42 ± 0.11	0.45 (0.2,0.65)	**0.53**
6, 7, 8, 9, 10, 11, 12			**0.520**
Horizontal coma.Pre	0.37 ± 0.11	0.39 (0.11,0.62)	**0.116**
Horizontal coma.3mo	0.38 ± 0.11	0.4(0.11,0.62)	
Horizontal coma.6mo	0.38 ± 0.12	0.4 (0.11,0.62)	**0.116**
Horizontal coma.12mo	0.38 ± 0.11	0.4 (0.11,0.62)	**0.055**
6, 7, 8, 9, 10, 11, 12			**0.895**
Horizontal trefoil.Pre	0.37 ± 0.11	0.35 (0.11,0.54)	**0.365**
Horizontal trefoil.3mo	0.37 ± 0.11	0.35(0.11,0.54)	
Horizontal trefoil.6mo	0.37 ± 0.11	0.39 (0.11,0.54)	**0.089**
Horizontal trefoil.12mo	0.37 ± 0.11	0.39 (0.11,0.54)	**0.5**
6, 7, 8, 9, 10, 11, 12			**0.894**
Vertical trefoil.Pre	0.36 ± 0.1	0.36 (0.11,0.54)	**0.020**
Vertical trefoil.3mo	0.37 ± 0.1	0.39(0.11,0.65)	
Vertical trefoil.6mo	0.36 ± 0.1	0.38 (0.11,0.6)	**0.114**
Vertical trefoil.12mo	0.36 ± 0.1	0.38 (0.11,0.6)	**0.160**
6, 7, 8, 9, 10, 11, 12			**0.571**
Spherical aberration.Pre	0.39 ± 0.08	0.41 (0.11,0.6)	**0.014**
Spherical aberration.3mo	0.4 ± 0.08	0.41(0.23,0.62)	
Spherical aberration.6mo	0.4 ± 0.08	0.41 (0.23,0.61)	**0.042**
Spherical aberration.12mo	0.39 ± 0.08	0.41 (0.11,0.6)	**0.109**
6, 7, 8, 9, 10, 11, 12			**0.4**
Total HOA. Pre	0.59 ± 0.13	0.6 (0.33,0.8)	**<0.001**
Total HOA.3mo	0.61 ± 0.13	0.63(0.33,0.9)	
Total HOA.6mo	0.61 ± 0.13	0.63 (0.33,0.9)	**0.005**
Total HOA.12mo	0.59 ± 0.13	0.6 (0.33,0.8)	**>0.999**
6, 7, 8, 9, 10, 11, 12			**0.005**

The average total higher-order aberrations showed a significant difference compared to before and between month 6 and 12 (P = 0.005).

No significant statistical difference was observed in the latest follow-up compared to before surgery.

In Table 2, the mean deviation (MD) and intraocular pressure (IOP) data of patients were provided and compared in different follow-up visits. In terms of statistics, there was no significant difference in IOP and changes in the visual field with the 24-2 strategy before and 12 months after surgery.

**Table 2:** Mean deviation and Intraocular pressure data of patients and comparison in different follow-ups.

Parameters	Mean ± SD	Median (Range)
MD.Pre	−0.56 ± 0.5	−0.5 (−1.99,0)
MD.3mo	−0.57 ± 0.49	−0.5(-1.96,0)
P-within	**0.850**	
MD.12mo	−0.56 ± 0.49	−0.5 (−1.96,0)
P-within	**0.605**	
IOP. Pre	14.5 ± 2.53	14 (10,21)
IOP.3mo	15.08 ± 7.89	14(10,11.1)
P-within	**0.373**	
IOP.12mon	14.5 ± 2.45	14 (10,21)
P-within	**0.913**	

MD: mean deviation, IOP: Intraocular pressure.

**Fig. 2:**
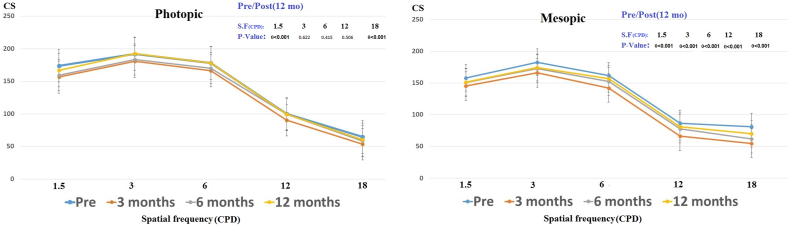
Contrast sensitivity under photopic and mesopic condition during follow up.

In Fig. 2, the information on contrast sensitivity photopic and mesopic condition of patients were presented and compared across different follow-ups. During the follow-up sessions, contrast sensitivity had initially decreased but improved over time. The contrast sensitivity has decreased across all spatial frequencies for mesopic conditions, and the decreases at 1.5 CPD and 18 CPD for photopic conditions were statistically significant. However, it still remains within the normal range.

Fig. 2 contrast sensitivity under photopic and mesopic condition during follow up.

The Table 3 shows a significant decrease in the changes of endothelial cells before and one year after surgery. Endothelial cell loss was 3.5 % one year after surgery and the changes in the Mean ± SD during the first, second and third trimesters, along with the coefficient of variation (CV) endothelial cell count(ECC) and hexagonality (HEX) in Table 4.

**Table 3:** The changes of endothelial cells before and one year after surgery.

Parameters	Mean ± SD	Median (Range)
ECC Pre	2393.29 ± 123.69	2410 (2100,2700)
ECC months 12	2308.58 ± 126.64	2308 (2001,2650)
P-within	<0.001	

ECC: Endothelial cell count.

**Table 4:** Changes in the coefficient of variation (CV), endothelial cell count (ECC), and hexagonality (HEX) are presented as Mean ± SD for the first, second, and third trimesters.

	First trimester	Second trimester	Third trimester
**Endothelial Cell Count(ECC)**			
Right	−62.52 ± 208.42	−10.35 ± 253.05	−19.25 ± 138.55
Left	−51.63 ± 109.99	−20.19 ± 134.69	−12.19 ± 84.89
**Coefficient of Variation (CV)**			
Right	12.39 ± 3.78	−6.42 ± 4.54	−2.7 ± 4.97
Left	12.48 ± 3.53	−6.9 ± 4.84	−2.02 ± 5.25
**percentage of Hexagonal Cells (HEX)**			
Right	−11.27 ± 6.10	3.77 ± 5.67	3.46 ± 5.33
Left	−9.48 ± 4.95	2.44 ± 5.17	2.45 ± 4.21

### 4.1 Complications and patient satisfaction

In this study, we did not observe any specific complications, such as inflammation, infection, uveitis, corneal perforation, neovascularization, increased IOP, retinal problems, or color fading that would require retouching. Two patients who had not paid attention to color selection underwent surgery again, and we changed the color. This change in color did not imply a dull or faded color. Only two cases of patients experienced severe light sensitivity and dry eye for four months, and their symptoms improved with medication.

**Fig. 3:**
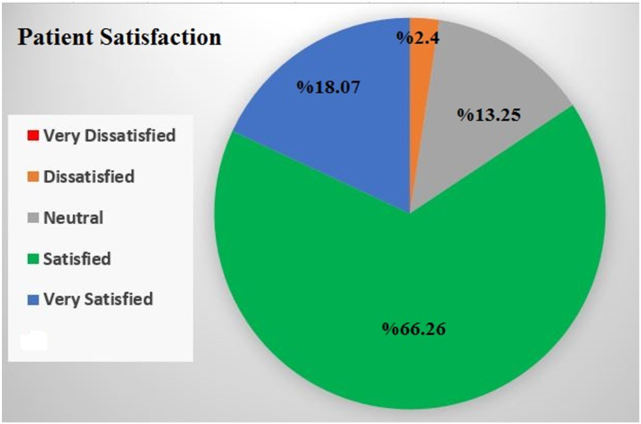
Patient satisfaction after keratopigmentation surgery after one year.

**Fig. 4:**
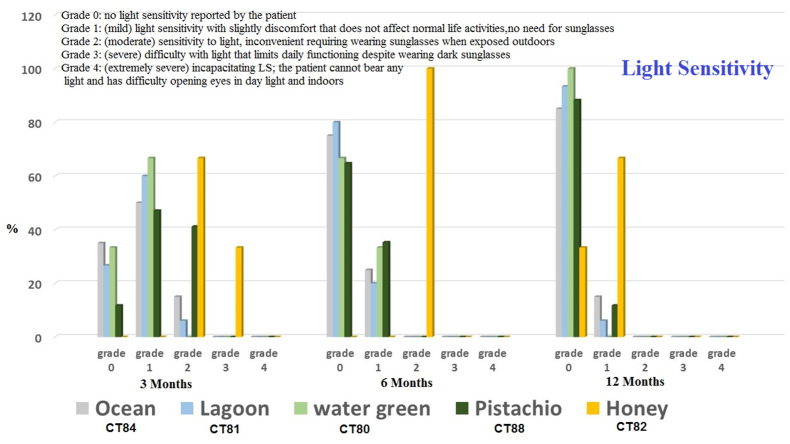
Light sensitivity after keratopigmentation surgery during follow up.

Patient satisfaction scores showed positive outcomes, with 84.33 % of patients reporting high satisfaction levels. (Fig. 3). Fig. 4 displays the usage of pigments, which include the following: 20 Ocean (CT84), 17 Pistachio (CT88), 15 Lagoon (CT81), 25 Emerald green (CT83), 3 Honey gold (CT82), and 3 Water green (CT80). In the latest follow-up, the light sensitivity (Grade 0) percentages were as follows: Ocean - 85 %, Lagoon - 93.33 %, Water Green - 100 %, Emerald Green - 76 %, Pistachio - 88.23 %, and Honey Gold - 33.33 %.

The Fig. 5 displays before and after surgery.

**The Fig. 5:**
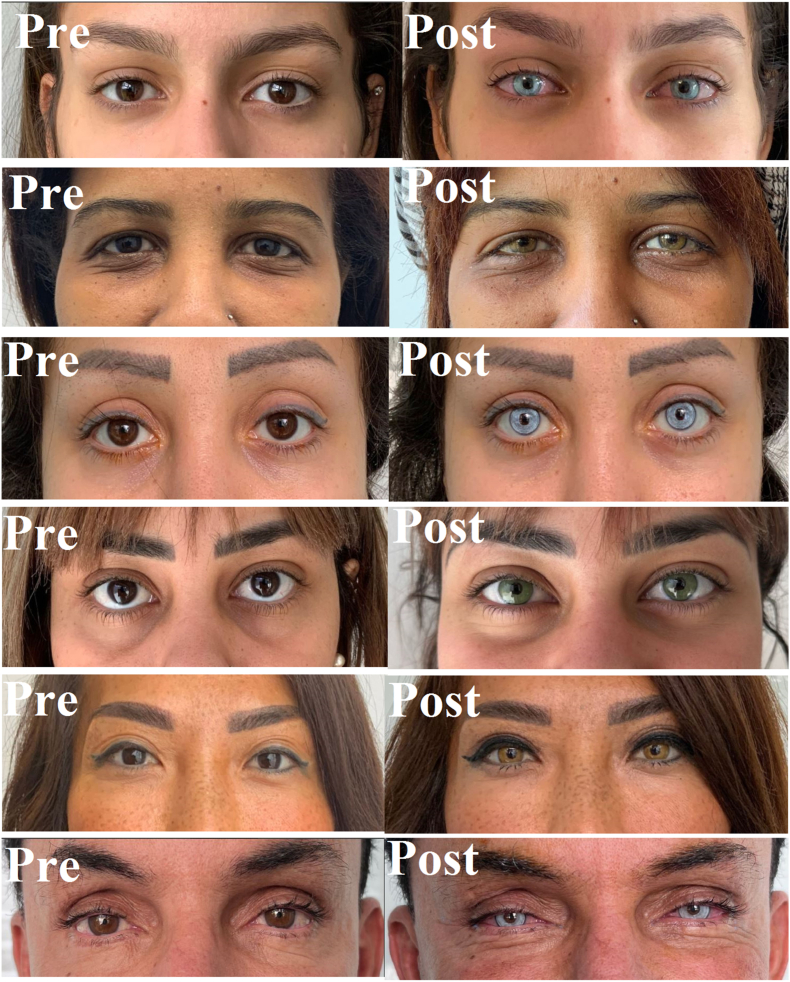
Displays before and after surgery.

## 5 Discussion

Keratopigmentation is a safe and effective technique for altering the color of the eye. It has been used successfully in patients with ocular trauma, aniridia, and congenital iris abnormalities.6-8 The use of this technique for cosmetic purposes is gaining popularity, but it should be noted that there are potential risks associated with the procedure, including infection, corneal haze, and decreased visual acuity.

Almost in all the studies conducted, visual acuity has not decreased and remained unchanged,^3^^,^^7^, ^8^, ^9^, ^10^. which in the present study, visual acuity did not change with or without correction.

Most of the studies conducted have been on damaged eyes. The first study on healthy eyes was conducted in 2001 by Alio et aland the first paper with a systematic analysis of the outcomes was published by the same author.3,7 which did not report any changes in the amount of refraction and astigmatism over a 2.5-year follow-up period. In another study conducted by D'Oria et al.^11^ in 2021, 79 healthy eyes underwent pure keratopigmentation but did not report significant changes in refraction in this study refractive changes (sphere and cylinder) in the one-year follow-up period, initially some hyperopic shifts were observed, but over time they returned to normal. In fact, because the corneal periphery is affected in this surgery, the central cornea becomes flatter, which is the reason for the hyperopic shifts. Although these changes occur two months after surgery and then return to their original state. Regarding the amount of astigmatism, since we have a 270-degree incision for injecting pigment in this surgery, it can have a slight effect on astigmatism, which was statistically significant in this study but not considered significant clinically.

In the present study, changes in IOP were not significant, and the reason for this may be that keratopigmentation surgery is an extraocular surgery. In other studies, that have been conducted previously, and where most of the patients under study had injured eyes such as iris defect, IOP may be affected due to the character disease. Therefore, it is not possible to compare their results with the results of healthy eyes.

Contrast sensitivity has statistically significantly decreased, but the results were within the normal range for all spatial frequencies. We are aware of justifying the reduction in contrast sensitivity regarding the decrease in quantity and quality of tear film, and previous studies have confirmed the relationship between dry eye and contrast sensitivity.^12^, ^13^, ^14^.

In our latest follow-up, we did not observe any statistically significant changes compared to before the surgery. when selecting the size of an artificial pupil on the cornea, we measure the actual size of the pupil in mesopic light condition. We set the size of the artificial pupil to be equal or 2 mm larger than the actual pupil size in mesopic conditions. By the way, considering the study conducted on Alafaleq et al.^15^ that demonstrated that a pupil size of 5 mm or larger does not impose any limitations on fundoscopy examination, it is better for the pupil size to be greater than 5 mm.

However, as before, the entrance light is adjusted based on the actual pupil size during surgery, and the artificial pupil has no effect on the incoming light to the eye so if in some patients the artificial pupil is not circular, it still may not have an effect on the incoming light, especially spherical aberration, even though González et al.^16^ demonstrated in his study that it is not necessary to have a circular aperture for good image quality.

The only study that has examined the visual field was conducted by Alio et al.7, who reported visual restrictions in 4 % of patients up to two months after surgery. In the current study, we examined the parameter mean deviation (MD) with Humphrey Field Analyzer with the 24-2 SITA and did not observe any restrictions, and did not have any reports of patient complaints regarding periphery visual field limitations (either due to patient adaptability or the use of larger artificial pupils). However, if we had examined the field of vision with the 60-2 strategy, we may have better-investigated the periphery visual field, which is one of the weaknesses of this study.

There have been few studies examining endothelial cells in healthy eyes because diseases affecting the eyes can impact the health of endothelial cells, making it difficult to compare results. A study conducted by Ferrari et al.^17^. (2018) found a 3 % decrease in endothelial cell count after one year. Similarly, our one-year results also demonstrated a 3 % decrease in endothelial cell count. parameters such as the coefficient of variation (CV), hexagonality (HEX), and pleomorphism are essential for a comprehensive evaluation. Our study acknowledges that endothelial cell patterns can vary significantly across neighboring areas and that repeated measurements can yield different results due to these inherent complexities. we performed an assessment 3,6 and 12 months' post-procedure, we recognize that intermediate measurements would have provided greater insight into the stability and progression of endothelial health over time. It should be noted that if the surgery time is reduced and the femtosecond laser is used to create a packet to the closer limbus, reducing the need for manual cutting, the amount of damage to corneal nerves and endothelial cells can be decreased.

A study conducted by Alafaleq et al.^18^ examined the level of satisfaction and safety in retouch surgery and reported a high level of satisfaction and safety in the surgery. The satisfaction rate in our study at the latest follow-up was 84.33 %, and 13.25 % of patients had a neutral opinion, mostly because they did not pay attention to color selection. Otherwise, a very high percentage of patients were satisfied. The correct color selection is the most important point in this surgery. Before the surgery, we test the colors on the face using computer software and reduce the intensity of each color by 30 %. Then we show it to the patient (based on our experience with the results of colors on patients who have previously undergone surgery and a long time has passed). With this approach, we prepare the patient's mind for color fading, and the patient sees the result of color fading on their own face. This may be a reason why we did not demand retouching in one year.

The first case report study on healthy eyes was conducted in 2014 by Dr. Ferrari, which examined only one patient. However, we analyzed a larger number of patients by assessing more parameters.^19^. By assessing these objective parameters, we can gain valuable insights into the safety and efficacy of pure keratopigmentation as a cosmetic procedure and provide evidence-based recommendations to patients considering keratopigmentation. The findings of this study will contribute to the existing literature and help guide clinicians and patients in making informed decisions regarding pure keratopigmentation for cosmetic purposes. Understanding the impact of pure keratopigmentation on visual function and ocular health is crucial for ensuring the long-term safety and success of this cosmetic intervention.

In a future study, we will separately investigate sensitivity to light, dry eye symptoms, the quality of tears, the level of color fading, and the possibility of washing for each pigment. Our recommendation is that further studies should be conducted on healthy eyes with a larger number of cases and more parameters to confirm the safety of the surgery.

In conclusion, Femtosecond laser-assisted keratopigmentation seems to be a safe and effective surgical technique for improving the pure cosmetic appearance of the eye. While the procedure may lead to a decrease in endothelial cell count, possibly related to the type of pigment use for this investigation, and contrast sensitivity, probably related to the restriction caused in the entrance of light through the neopupil, these changes are within acceptable limits and can be closely monitored in the follow-up period. Further studies with longer follow-up periods are required to confirm the long-term safety and efficacy of this procedure.

## CRediT authorship contribution statement

**Jorge Alio:** Writing – review & editing, Writing – original draft, Resources, Project administration, Methodology, Investigation, Funding acquisition, Formal analysis, Conceptualization. **Azad Sanginabadi:** Writing – original draft, Visualization, Validation, Supervision, Software, Resources, Investigation, Funding acquisition, Formal analysis, Data curation, Conceptualization. **Amir Theodore Hojabr:** Writing – review & editing, Writing – original draft, Visualization, Validation, Supervision, Project administration, Methodology, Investigation, Formal analysis, Data curation, Conceptualization. **Behzad Jafari:** Writing – review & editing, Software, Resources, Project administration, Investigation, Funding acquisition.

## Patient consent

The patients have given consent to publish this case.

## Funding

No funding or grant support.

## Declaration of competing interest

None of the authors has financial or proprietary interests in any of the materials or methods mentioned.
